# Phase II study of S-1 combined with oxaliplatin as therapy for patients with metastatic biliary tract cancer: influence of the *CYP2A6* polymorphism on pharmacokinetics and clinical activity

**DOI:** 10.1038/bjc.2011.17

**Published:** 2011-02-15

**Authors:** K-p Kim, G Jang, Y S Hong, H-S Lim, K-s Bae, H-S Kim, S S Lee, J-G Shin, J-L Lee, M-H Ryu, H-M Chang, Y-K Kang, T W Kim

**Affiliations:** 1 Department of Oncology, Asan Medical Center, University of Ulsan College of Medicine, 86 Asanbyeongwon-gil, Songpa-gu, Seoul 138-736, Korea; 2 Department of Clinical Pharmacology, Asan Medical Center, University of Ulsan College of Medicine, Seoul, Korea; 3 Department of Pharmacology and PharmacoGenomics Research Center, Inje University College of Medicine and Clinical Pharmacology Center, Busan Paik Hospital, Busan, Korea

**Keywords:** CRPC, dexamethasone, diethylstilbestrol, treatment sequencing

## Abstract

**Background:**

Advanced biliary cancer is often treated with fluoropyrimidine-based chemotherapy. In this study, we evaluated the efficacy and tolerability of a combination of S-1, an oral fluoropyrimidine prodrug, and oxaliplatin in patients with metastatic biliary cancer.

**Methods:**

Patients with histologically confirmed metastatic biliary cancer and no history of radiotherapy or chemotherapy were enrolled. Oxaliplatin was administered intravenously (130 mg m^−2^), followed by 14-day administration of oral S-1 (40 mg m^−2^ twice daily) with a subsequent 7-day rest period every 21 days. Pharmacokinetic analysis of S-1 was performed at cycle 1. Patients were genotyped for *CYP2A6* polymorphisms (^*^1, ^*^4, ^*^7, ^*^9 or ^*^10), and pharmacokinetic and clinical parameters compared according to the *CYP2A6* genotype.

**Results:**

In total, 49 patients were evaluated, who received a median of four cycles. The overall response rate was 24.5%. Median progression-free and overall survival was 3.7 and 8.7 months, respectively. The most common haematological grade 3 out of 4 toxicity was neutropenia (14%), while non-hematological grade 3 out of 4 toxicities included anorexia (14%), nausea (12%), asthenia (10%), vomiting (10%), and diarrhoea (4%). Biotransformation of S-1 (AUC_0−24 h_ of 5-fluorouracil/AUC_0−24 h_ of tegafur) was 1.85-fold higher for the *1/*1 group than for the other groups (90% confidence interval 1.37–2.49). Diarrhoea (*P*=0.0740), neutropenia (*P*=0.396), and clinical efficacy (response rate, *P*=0.583; PFS, *P*=0.916) were not significantly associated with *CYP2A6* genotype, despite differences in 5-FU exposure.

**Conclusion:**

The combination of S-1 and oxaliplatin appears to be active and well tolerated in patients with metastatic biliary cancer, and thus is feasible as a therapeutic modality. *CYP2A6* genotypes are associated with differences in the biotransformation of S-1. However, the impact of the *CYP2A6* polymorphism on variations in clinical efficacy or toxicity requires further evaluation.

Biliary tract cancer (BTC), including cancer of the gallbladder and the intrahepatic and extrahepatic bile ducts, is one of the most aggressive human malignancies. Gemcitabine and fluoropyrimidine constitute the backbone of palliative chemotherapy (Hezel and Zhu, 2008). 5-Fluorouracil (5-FU) displays clinical activity, both alone and in combination with platinum-based agents, in single arm phase II studies (Ducreux *et al*, 1998; Choi *et al*, 2000). Oral fluoropyrimidine derivatives, including capecitabine and uracil-tegafur (UFT), have been shown to be active as single agents, as well as in conjunction with cisplatin (Kim *et al*, 2003; Ikeda *et al*, 2005; Park *et al*, 2005).

S-1, an oral prodrug of 5-FU composed of tegafur, 5-chloro-2, 4-dihydroxypyridine (CDHP; gimestat), and potassium oxonate (otastat), shows clinical efficacy in patients diagnosed with various solid tumours, including biliary tract cancers (Park *et al*, 2009; Sasaki *et al*, 2009). Combinations of S-1 and cisplatin have also yielded modest response rates and tolerable toxicity profiles (Kim *et al*, 2008b; Kang *et al*, 2010).

The pharmacokinetic and toxicity profiles of S-1 differ with ethnicity (Hirata *et al*, 1999; Hoff *et al*, 2003; Chu *et al*, 2004). The principal dose-limiting toxicities are diarrhoea in Caucasian and myelosuppression in Asian patients (Hirata *et al*, 1999; Cohen *et al*, 2002). These differences may be attributable to genetic variations in *CYP2A6* encoding a cytochrome P450 enzyme that has a major role in the biotransformation of tegafur to 5-FU (Ikeda *et al*, 2000). One clinical study demonstrated an association of the CYP2A6*4 allele with exposure to 5-FU in non-small-cell lung cancer patients receiving S-1, while another study suggested that exposure to CDHP, which affects the metabolism of 5-FU, is the key determinant of variability in 5-FU efficacy following S-1 administration (Fujita *et al*, 2008; Kaida *et al*, 2008).

The primary objective of the present study was to evaluate the efficacy and safety of S-1 in combination with oxaliplatin chemotherapy in patients with metastatic BTC and objective response rate was the primary endpoint. The secondary objective was to evaluate the relationships between pharmacokinetics, clinical activity and tolerability of S-1-based chemotherapy and *CYP2A6* genotypes.

## Patients and Methods

### Eligibility criteria

Patients with histologically confirmed recurrent or metastatic cancer of the biliary tract were eligible, provided that they met the following criteria, including at least one unidimensionally measurable lesion according to the Response Evaluation Criteria in Solid Tumours (RECIST) guidelines Version 1.0, age between 18 and 70 years, Eastern Cooperative Oncology Group (ECOG) performance status of 0–2, adequate haematological (haemoglobin ⩾9.0 g dl^−1^, absolute neutrophil count (ANC) ⩾1.5 × 10^9^ l^−1^, and platelet count ⩾100 × 10^9^ l^−1^), hepatic (total bilirubin ⩽1.5 mg dl^−1^, serum transaminase ⩽3 × upper normal limit or ⩽5 × upper normal limit in cases of hepatic metastases), and renal (serum creatinine ⩽1.5 mg dl^−1^) functions, no history of radiation therapy or chemotherapy, including S-1 or oxaliplatin treatment within 6 months, and no history of gastrointestinal operations, including gastrectomy or pancreaticoduodenectomy. Patients were excluded if they had central nervous system metastasis, gastrointestinal obstruction, bleeding or sensory neuropathy greater than grade 2, according to the National Cancer Institute of Common Toxicity Criteria (NCI CTC) Version 3.0. Creatinine clearance was calculated using the Cockcroft–Gault equation (Cockcroft and Gault, 1976). The protocol was approved by the institutional ethics committee, and all patients provided written informed consent before enrollment. This study was conducted using the Good Clinical Practice guidelines, in accordance with the Declaration of Helsinki and its amendments.

### Study design and treatment

This was a single centre phase II study performed to evaluate the efficacy and safety of the S-1 and oxaliplatin combination therapy. S-1 was administered orally at a dose of 40 mg m^−2^ twice daily for 14 days, followed by a 7-day rest period. S-1 doses were calculated in milligrams per square metre of body surface area, and rounded down to the nearest 5 or 10 mg. For pharmacokinetic analysis, S-1 was given only once on day 1 of cycle 1. Beginning on day 2, S-1 was administered twice daily. Oxaliplatin was administered intravenously at 130 mg m^−2^ for 2 h on day 2 of cycle 1, and day 1 of cycle 2 and subsequent cycles. Treatment was repeated every 21 days, and continued for a maximum of 9 cycles in the absence of progressive disease, development of unacceptable toxicity, or patient refusal.

Doses of S-1 and oxaliplatin were reduced based on the extent of haematologic and non-hematologic toxicities evaluated before each treatment cycle, according to NCI CTC toxicity scale Version 3.0. At the first occurrence of grade 3 thrombocytopenia or grade 4 neutropenia lasting <5 days, S-1 treatment was interrupted, but was resumed at the same dose after resolution to grade 1 or better. At the second or third occurrence of the same haematologic toxicity with similar severity, treatment was interrupted, followed by 25 or 50% dose reduction of S-1 and oxaliplatin (to 100 and 85 mg m^−2^, respectively) in subsequent cycles. If the haematologic toxicity recurred with similar severity after adequate dose reduction, treatment was discontinued. In cases of grade 3 or 4 non-hematologic toxicity, treatment was interrupted, followed by a 25 or 50% dose reduction, respectively. The oxaliplatin dose was modified in cases where patients developed grade 2 neurosensory toxicity. Treatment was interrupted until toxicity resolved to grade 1 or better, with reduction of the oxaliplatin dose to 100 mg m^−2^ in subsequent cycles. In patients experiencing a second occurrence of grade 2 neurosensory toxicity, treatment was interrupted, and the oxaliplatin dose reduced to 85 mg m^−2^. Treatment was discontinued if patients experienced recurrence of grade 2 neurosensory toxicity after two oxaliplatin dose reductions, if oxaliplatin therapy was delayed for >2 weeks because of toxicity, or if patients developed grade 3 or higher neurosensory toxicity.

### Efficacy and safety assessments

All patients who received at least one cycle of treatment were subjected to efficacy and safety assessments. Response rate, the primary endpoint, was evaluated according to the Response Evaluation Criteria in Solid Tumours (RECIST) Version 1.0 (Therasse *et al*, 2000). Evaluation of tumours was performed every two cycles. Chemotherapy was discontinued in cases of disease progression, unacceptable toxicity or withdrawal of consent. Adverse drug reactions were graded according to National Cancer Institute Common Toxicity Criteria Version 3.0.

### Pharmacokinetic assay

Blood samples (about 10 ml) were collected in heparinised tubes at 0.5, 1, 2, 4, 8, 12, and 24 h after the first single dosage of S-1 administered during cycle 1. Tubes were centrifuged (3500 r.p.m., 8 min, 4°C), and plasma immediately separated and transferred as three aliquots into storage cryotubes, which were frozen at −80°C until analysis. Plasma concentrations of all compounds were determined using liquid chromatographic–tandem mass spectrometry. Tegafur, FU, and CDHP were analysed on the API 3000 LC/MS/MS system (MDS Sciex, Concord, Ontario, Canada). Plasma concentration data were used to calculate pharmacokinetic parameters, including area under the curve from 0 to 24 h (AUC_0−24 h_) and maximum concentration (C_max_), by the linear trapezoid method using WinNonlin (Professional Network Version 5.2; Pharsight Corporation, Mountain View, CA, USA).

### *CYP2A6* genotyping assay

Genomic DNA was extracted from whole blood using a specific DNA preparation kit (QIAamp Blood Mini Kit; Qiagen, Valencia, CA, USA), according to the manufacturer's instructions. Genotypes of CYP2A6^*^1, ^*^7, ^*^9 and ^*^10 allelic variants were determined using the multiplex minisequencing (SNaPshot, ABI PRISM SNaPshot Multiplex Kit; Applied Biosystems, Foster City, CA, USA) method. Briefly, the full-length *CYP2A6* gene was amplified using a pair of primers, specifically, 5′-CTCTCCCCTGGAACCCCCAG-3′ and 5′-GCACTTATGTTTTGTGAGACATCAGAGACAA-3′. PCR was performed on a 9700 thermal cycler (PE Applied Biosystems, Foster city, CA, USA) under the following conditions: initial denaturation at 94°C for 1 min, followed by 30 cycles of 98°C for 20 s, 64°C for 30 s, 72°C for 3 min 30 s, and a final elongation step at 72°C for 10 min. The 7.5 kb PCR product was used as a template for the SNaPshot reaction, which contained the following sequencing primers: *CYP2A6*-48T>G (^*^9), 5′-GGCTGGGGTGGTTTGCCTTT-3′ *CYP2A6* 6600G>T (^*^10), 5′-GGAAGCTCATGGTGTAGTTT-3′ and *CYP2A6* 6558T>G (^*^7), 5′-CTCCCAGTCACCTAAGGACA-3′. Genotype analyses were performed with GeneMapper (Version 3.7) software (Applied Biosystems). For genotyping the *CYP2A6* deletion allele (^*^4), allele-specific amplification and restriction fragment length polymorphism analysis were performed, as described previously (Nakajima *et al*, 2001).

### Statistical analyses

Standard descriptive and analytical methods were used to describe the patient population and their baseline characteristics. This trial was designed using Fleming's single-stage Phase II procedure to test the null hypothesis that the true objective response rate was <15%, with a sample size sufficient to reject it when the true objective response rate was >35% with an 80% power and using a level of *α*=5% (one-sided test), and required a sample size of 44 patients (Machin *et al*, 1997; Kim *et al*, 2008b). We assumed a dropout rate of 10%, making the required number of patients 49.

To analyse the effects of *CYP2A6* genotypes, the pharmacokinetic parameters of S-1 were log-transformed and compared using mixed model analysis of variance (ANOVA). To evaluate *CYP2A6* genotype-drug interactions, the differences in log-transformed mean values between wild type (^*^1/^*^1) and variants (single or double) were calculated, and both point estimates and 90% confidence interval (CI) back-transformed to obtain geometric mean ratios, along with CI values for the ratios. Progression-free survival (PFS) was calculated from the commencement of chemotherapy to date of disease progression or death from any cause, and overall survival (OS) calculated from the start of chemotherapy to date of death from any cause. Survival analyses were performed using the Kaplan–Meier method with the log-rank test. Data analyses were performed using the Statistical Software Package for Social Sciences (SPSS version 14.0; Chicago, IL, USA).

## Results

### Patient characteristics

From September 2006 to January 2008, we enrolled a total of 49 patients with histologically confirmed recurrent or metastatic adenocarcinoma of the biliary tract (Table 1). No patient had previously received radiation therapy or chemotherapy, including S-1 or oxaliplatin. Median patient age was 52 years (range, 23–68 years), and 31 patients (63%) were male. Most patients (94%) showed good performance status with ECOG scale values of 0–1. All patients had good renal function, and mean creatinine clearance was 97.40 ml min^−1^ (range, 57.13–198.0 ml min^−1^). A total of 30 patients (61%) were diagnosed with intrahepatic cholangiocarcinoma, and 10 (20%) with gallbladder cancer. In all, 40 patients (82%) had metastatic abdominal lymphadenopathy, with the liver being the most common metastatic organ (67%).

### Efficacy

**Figure 1:**
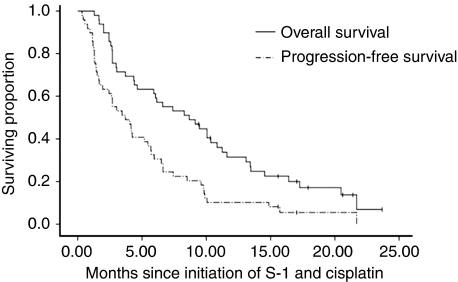
Kaplan–Meier curves of progression-free survival (PFS) and overall survival (OS) for all 49 patients. Median PFS and OS were 3.7 months (95% CI: 1.7–5.7 months) and 8.7 months (95% CI: 4.8–12.6 months).

Among the 49 study patients, 45 were evaluated for response. The remaining four subjects were lost to follow-up and hence were not assessable. Partial response was observed in 24.5% (12 out of 49) and stable disease in 34.7% (17 out of 49) of patients. The overall response rate in the intention-to-treat population was 24.5% (95% (CI), 12.5–36.5% Table 2). Patients with gallbladder cancer tended to show a higher response rate than those with intra- or extra-hepatic cholangiocarcinoma, but the difference was not statistically significant. At a median follow-up duration of 20 months (range, 9.4–25.6 months), median PFS and OS for all patients were 3.7 months (95% CI: 1.7–5.7 months) and 8.7 months (95% CI: 4.8–12.6 months), respectively (Figure 1). We observed no significant survival differences with respect to tumour location (data not shown).

### Drug exposure and safety

The 49 patients underwent a total of 216 treatment cycles (median, 4 cycles), 212 of which were assessable for toxicity. The frequencies of haematological and non-haematological toxicities are summarised in Table 3. The most common (>5%) grade 3 out of 4 haematological toxicity was neutropenia with an incidence of 12% (6 out of 49). However, no febrile neutropenia was observed. The most common non-hematological grade 3 out of 4 toxicity was anorexia (7 out of 49, 14%), followed by nausea (6 out of 49, 12%), vomiting (5 out of 49, 10%), asthenia (2 out of 49, 4%), stomatitis (2 out of 49, 4%), and diarrhoea (2 out of 49, 4%).

A total of 54 treatment cycles (26%, 54 out of 207 cycles) were delayed in 24 patients. Chemotherapy doses were reduced for 65 out of 207 cycles (31%) in 26 patients. The median dose intensities were 315.1 mg m^−2^ per week (range, 106.4–373.3 mg m^−2^ per week) for S-1 and 39.4 mg m^−2^ per week (range, 24.2–43.3 mg m^−2^ per week) for oxaliplatin, with relative dose intensities of 84.4 and 91.1%, respectively.

### Relationships among pharmacokinetic parameters, clinical efficacy, toxicity of S-1, and *CYP2A6* genotypes

*CYP2A6* genotypes were analysed in all 49 patients, and full pharmacokinetic data were obtained for 48 of the patients. Wild-type homozygotes (CYP2A6*1/*1) were detected in 14 patients, and single and double variants in 28 and 7 patients, respectively. The allele frequencies of CYP2A6*4, *7, *9, and *10 were 10.2, 8.2, 19.4, and 4.1%, respectively. These values did not deviate significantly from the Hardy–Weinberg equilibrium.

**Figure 2:**
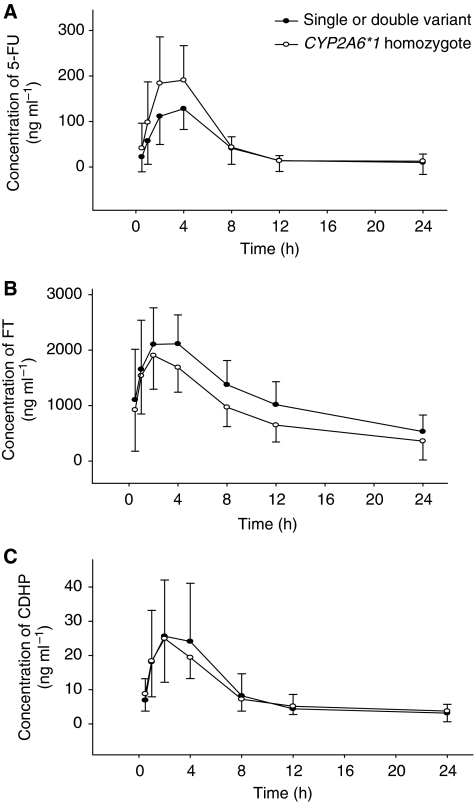
Mean concentration–time curves of 5-FU (**A**), FT (tegafur, **B**), and CDHP (**C**) in 48 patients treated with S-1 and oxaliplatin.

In 48 patients, the 5-FU level reached peak plasma concentrations (C_max_) about 4 h after administration of S-1. The pharmacokinetic parameters are presented in Table 4. Plasma concentrations of 5-FU tended to be higher in the CYP2A6 *1 homozygotes than patients with variant gene types (Figure 2). Specifically, C_max, 5−FU_ and AUC_0−24 h, 5−FU_ for *CYP2A6* variant types were 70% (90% CI: 55–88%) and 74% (90% CI: 56–98%) those for *1 homozygotes (Table 4). On the other hand, FT plasma concentrations were lower in the CYP2A6 *1 homozygotes, compared with those in patients with genetic variations (Figure 2); The C_max,FT_ and AUC_0−24 h,FT_ values for CYP2A6 *1 homozygotes were 17% (90% CI: 2–33%) and 37% (90% CI: 19–58%) higher than those with variant genes (Table 4). CDHP plasma concentrations were similar in both groups.

**Figure 3:**
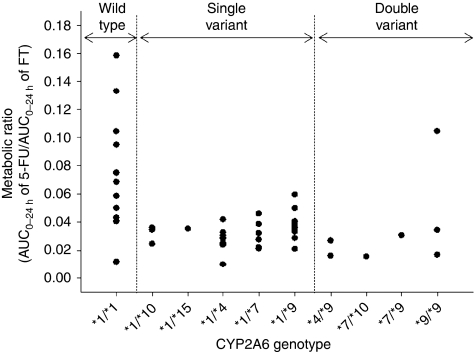
Comparison of metabolic ratios based on type of *CYP2A6* polymorphism in 48 patients treated with S-1 and oxaliplatin. Genotypes were categorised according to the number of variant (non-wild type) alleles.

**Figure 4:**
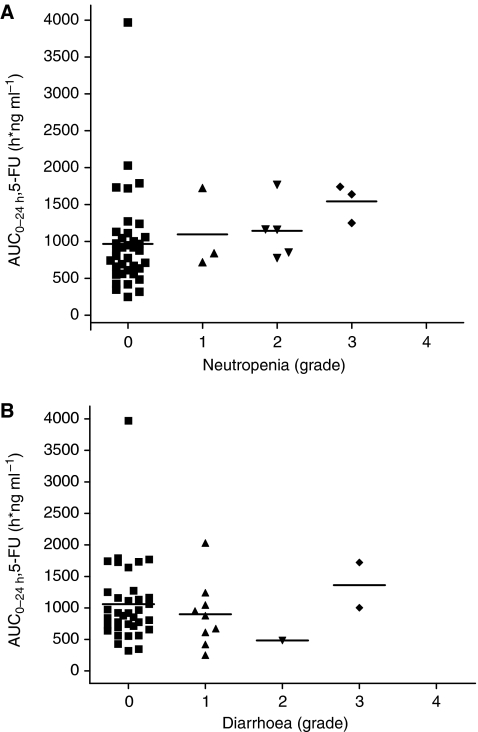
Relationship between exposure to 5-FU and severity of toxicity for the first cycle in 48 patients (expressed as dots) treated with S-1 and oxaliplatin; (**A**) neutropenia (**B**) diarrhoea.

The ratio between exposure to 5-FU and FT (AUC_0−24 h_ of 5-FU/ AUC_0−24 h_ of FT) was 0.0710±0.0433 in the wild-type group and 0.0331±0.0160 in patients with single or double variants (Table 4, Figure 3), yielding a 1.85-fold higher metabolic ratio (90% CI: 1.37–2.49) in the wild-type group. However, comparison of the frequencies of diarrhoea and neutropenia between wild type and variants revealed that the CYP2A6 *1/*1 genotype was not significantly associated with any grade of diarrhoea (wild type, 5 out of 13, *vs* variant type, 7 out of 35; *P*=0.0740) or neutropenia (wild type, 4 out of 13, *vs* variant type, 7 out of 35; *P*=0.396). In addition, exposure to 5-FU (AUC_0−24 h, 5−FU_) was not significantly correlated with severity of diarrhoea or neutropenia (Figure 4).

## Discussion

This study evaluated the efficacy and safety of S-1 and oxaliplatin in Korean patients with metastatic biliary tract carcinoma. The response rate was 24.5%, and median PFS and OS were 3.7 and 8.7 months, respectively, similar to results obtained from trials of S-1 combinations with cisplatin and oxaliplatin (Kim *et al*, 2008b; Oh *et al*, 2008). The frequencies of diarrhoea (4%) and neutropenia (14%) exceeding grade 3 were comparable with those of other studies, which used similar dosages of S-1 and oxaliplatin (Yamada *et al*, 2008; Zang *et al*, 2009).

Recently, survival gain in biliary tract cancers with gemcitabine-based chemotherapy was reported. Moreover, a combination of gemcitabine and cisplatin displayed benefits over gemcitabine monotherapy in a randomised, phase III trial (Valle *et al*, 2010). A small-scale randomised phase II trial showed that survival rates with gemcitabine and oxaliplatin surpassed those with fluorouracil and folinic acid in unresectable gall bladder cancer (Sharma *et al*, 2010). On the other hand, several phase II trials have supported the utility of 5-FU-based containing regimens for biliary tract cancers (Eckel and Schmid, 2007). Although gemcitabine-based chemotherapy may be the preferred treatment based on recent phase III trials, it is not evident whether gemcitabine surpasses fluoropyrimidine in terms of efficacy (Kim *et al*, 2008a). Combination chemotherapy consisting of gemcitabine, 5-FU derivatives and platinum analogue regimens led to response rates between 20 and 30% and median survival of 8–12 months (Verslype *et al*, 2008).

The recorded overall response of 24.5% meant that our study did not meet its primary endpoint response rate of 35%. However, some biologic features of biliary tract cancers should be considered. First, response rates and survival differ significantly according to the primary site of the tumour, and intrahepatic cholangiocarcinoma has the worst prognosis (Park *et al*, 2009). The proportion of intrahepatic cholangiocarcinoma was substantially higher than expected (61%), which may have influenced the overall response rate. Second, many studies have used tumour control rate (defined as stable disease, complete or partial response) to assess efficacy owing to the aggressive nature of advanced biliary tract cancer. We recorded a tumour control rate of 59.2%, which is comparable with data from previous studies with S-1 and cisplatin (Kim *et al*, 2008b). Third, we recently showed that gemcitabine was better than S-1 as a doublet partner for platinum agents in advanced biliary cancers (Kang *et al*, 2010). However, gemcitabine was associated with higher toxicities, especially neutropenia and thrombocytopenia. As fluoropyrimidine-based chemotherapy induces lower haematological toxicities and decreased tendency of biliary tract cancer-related infections, including cholangitis and liver abscess, S-1 use may be favored in a subset of patients (Kim *et al*, 2008a). Moreover, S-1 offers the convenience of oral administration, in contrast to the weekly intravenous administration of gemcitabine. Therefore, well-designed randomised phase III trials are required to compare fluoropyrimidine plus platinum combination with gemcitabine plus platinum combination chemotherapy in terms of efficacy and safety.

Despite these advantages, S-1 pharmacokinetics is subject to interindividual variability, which is explained by the pharmacogenetics of CYP2A6 (Goh *et al*, 2008). The variant genotypes of CYP2A6 display decreased baseline enzyme activity, leading to reduced 5-FU formation. We categorised pharmacokinetic parameters as ‘wild type’ (^*^1/^*^1) and ‘variant type’ (single and double variant) genotypes, consistent with pharmacogenetic studies on nicotine, another substrate of CYP2A6 (Schoedel *et al*, 2004; Nakajima *et al*, 2006; Mwenifumbo *et al*, 2007). On comparing the ratio between AUC_0−24 h_ of 5-FU and AUC_0−24 h_ of FT by *CYP2A6* genotype, this ratio was 1.85-fold greater in the*1/*1 group than the variant-type group. A previous study reported that non-small cell lung cancer patients with CYP2A6*4 alleles, which complete lack relevant enzymatic activity, show significantly lower plasma concentrations of 5-FU (Kaida *et al*, 2008). In addition to CYP2A6*4 alleles, CYP2A6*7, *9, and *10 displayed decreased enzyme activity. Our present observations have expanded the application of *CYP2A6* genotyping by including various alleles in the interpretation of S-1 pharmacokinetics.

Despite differences in exposure, the *CYP2A6* genotype was not significantly associated with clinical efficacy or incidence of toxicity, suggesting that the pharmacokinetic-pharmacodynamic relationships of 5-FU are complicated. Studies using various schedules and formulations of 5-FU have reported a correlation between exposure to 5-FU and toxicity, particularly diarrhoea (Cohen *et al*, 2002; Chu *et al*, 2004; Ajani *et al*, 2005; Gusella *et al*, 2006; Gamelin *et al*, 2008). Conversely, other studies have been unable to establish an association between the AUC of 5-FU and toxicity after S-1 administration (Hirata *et al*, 1999). This lack of association may also be attributed to the other constituents of S-1. The action of oxonate, which inhibits the orotate phosphoribosyl-transferase (OPRT) enzyme in intestinal tissue and decreases the occurrence of diarrhoea, may be related to interindividual differences (Shirasaka *et al*, 1993; Peters *et al*, 2003). The clinical efficacy of fluoropyrimidines, including S-1, is influenced by thymidylate synthase, which may confound the association between 5-FU exposure and clinical efficacy (Ichikawa *et al*, 2004). A recent investigation reported a correlation between the *CYP2A6* genotype and efficacy of S-1-based chemotherapy in advanced gastric cancer (Kong *et al*, 2009). However, pharmacokinetic data for clarifying the exposure-effect relationship were not evaluated.

S-1 is mainly used in Korea and Japan, whereas capecitabine is often used in Western countries. In a study on the efficacy of capecitabine and oxaliplatin in advanced biliary tract cancers, Nehls *et al* (2008) observed a tumour control rate of 77%, which is higher than that obtained in our study. However, it must be noted that patient characteristics between the two studies differed, since we analysed more cases of intrahepatic cholangiocarcinoma, which has the worst prognosis among the biliary tract cancers. An additional finding is that the recommended dose of S-1 for Asians is higher than that for Caucasians (Hoff *et al*, 2003). This has been explained by the fact that Caucasians show a higher frequency of wild-type CYP2A6 and suffer from gastrointestinal toxicities. Another Korean group recently reported adequate efficacy and safety with 50 mg m^−2^ S-1 twice a day in combination with 130 mg m^−2^ oxaliplatin for colorectal cancer (Kim *et al*, 2009).

In conclusion, the present results suggest that a combination of S-1 and oxaliplatin is active and safe for patients with advanced biliary tract cancer. At present, gemcitabine is favored as the backbone for chemotherapy in biliary tract cancers, based on efficacy studies. However, an oral 5-FU based regimen, such as capecitabine or S-1, may be preferable in terms of convenience. Large-scale studies are required to properly address this issue. Our findings further indicate that differences in the *CYP2A6* genotype may affect individual pharmacokinetics of S-1, but do not have a significant impact on clinical efficacy or toxicity.

## Figures and Tables

**Table 1:** Patient characteristics (*n*=49)

**Characteristic**	**No. of patients (%)**
Median age, year (range)	52 (23–68)
	
*Gender*	
Male	31 (63.3)
Female	18 (36.7)
	
*ECOG performance status*	
0	25 (51.0)
1	23 (46.9)
2	1 (2.1)
	
*Location of primary tumour*	
Gallbladder	10 (20.4)
Intrahepatic bile duct	30 (61.2)
Extrahepatic bile duct	9 (18.4)
	
*Tumour differentiation*	
Well differentiated	5 (10.2)
Moderately differentiated	31 (63.3)
Poorly differentiated	10 (20.4)
Unknown	3 (6.1)
	
*Disease status*	
Metastatic	32 (65.3)
Recurrent	17 (34.7)
	
*Metastatic sites*	
Liver	33 (67.3)
Lung	12 (24.5)
Cervical node	2 (4.1)
Abdominal node	40 (81.9)
Peritoneum	19 (38.8)
Bone	2 (4.2)
Others	4 (8.2)
	
*No. of metastatic organs*	
1	14 (28.6)
2	18 (36.7)
3	11 (22.4)
>3	6 (12.2)
	
*CA 19-9*	
Elevated	35 (71.4)
Normal	14 (28.6)
	
*CYP2A6 genotype* a	
1 homozygote^a^	13 (27.1)
Single variant	28 (58.3)
Double variant	7 (14.6)

a *CYP2A6* genotypes were performed in 48 patients.

**Table 2:** Treatment response (intention-to-treat analysis, *n*=49)

**Response**	**Gallbladder cancer (*n*=10)**	**Intrahepatic CCC (*n*=30)**	**Extrahepatic CCC (*n*=9)**	**All patients (*n*=49)**
CR	—	—	—	—
PR	4 (40.0%)	7 (23.3%)	1 (11.1%)	12 (24.5%)
SD	4 (40.0%)	9 (30.0%)	4 (44.4%)	17 (34.7%)
PD	—	12 (40.0%)	4 (44.4%)	16 (32.7%)
NE^a^	2 (20.0%)	2 (6.7%)	—	4 (8.2%)

Abbreviations: CR=complete response; PR=partial response; SD=stable disease; PD=progressive disease; NE=not evaluable; CCC=cholangiocellular carcinoma.

a Four patients were lost to follow-up.

**Table 3:** Common (>5%) haematological and non-haematological toxicities (intention-to-treat analysis)

	**Toxicity per cycle (*N*=212)**				**Toxicity per patient (*N*=49)**			
**Adverse events**	**Grade 1**	**Grade 2**	**Grade 3**	**Grade 4**	**Grade 1**	**Grade 2**	**Grade 3**	**Grade 4**
Anaemia	57	14	2	4^a^	28	7	1	2^a^
Leukopenia	18	18	—	—	9	9	—	—
Neutropenia	12	14	14	—	6	7	6	—
Thrombocytopenia	51	22	6	—	27	11	3	—
								
Asthenia	43	49	10	—	19	21	2	—
Anorexia	57	12	14	—	29	6	7	—
Nausea	55	14	12	—	25	7	6	—
Vomiting	22	8	10	—	11	4	5	—
Stomatitis	39	2	2	—	20	2	2	—
Diarrhoea	33	6	4	—	15	3	2	—
Constipation	49	6	—	—	21	4	—	—
Neuropathy	88	2	—	—	41	2	—	—
Hand-foot syndrome	18	2	—	—	9	1	—	—

a Two patients experienced tumour bleeding.

**Table 4:** Pharmacokinetic parameters of 5-FU, FT, and CDHP according to *CYP2A6* genotype

	*CYP2A6* *1 homozygote	**Single-variant of *CYP2A6***	**Double-variant of *CYP2A6***	**Point estimate ratio (90% confidence interval)** a
*5-FU*				
C_max_ (ng ml^−1^)	218.1±95.88	144.5±43.35	138.1±70.03	0.70 (0.55–0.88)
AUC_0–24 h_ (hng ml^−1^)	1295±590.4	875.5±303.0	1154±1262	0.74 (0.56–0.98)
				
*FT*				
C_max_ (ng ml^−1^)	2095±497.0	2398±559.1	2691±948.2	1.17 (1.02–1.33)
AUC_0–24 h_ (hng ml^−1^)	20770±6831	27060±5101	32380±14650	1.37 (1.19–1.58)
				
*CDHP*				
C_max_ (ng ml^−1^)	28.23±10.44	30.32±15.62	39.73±24.55	1.06 (0.80–1.40)
AUC_0–24 h_ (hng ml^−1^)	203.4±46.83	213.5±125.6	208.1±132.4	0.93 (0.72–1.20)
				
*5-FU/FT*				
C_max_	0.1083±0.0494	0.0618±0.0195	0.0526±0.0205	1.67 (1.31–2.13)
AUC_0–24 h_	0.0710±0.0433	0.0327±0.0100	0.0348±0.0315	1.85 (1.37–2.49)

Abbreviations: FT=tegafur; C_max_=maximum plasma concentration; AUC_0–24 h_=area under the plasma concentration-time curve from time 0–24 h; 5-FU/FT=ratio between the pharmacokinetic parameters of 5-FU and FT.

a Point estimate ratio between single or double variant and *1 homozygote. Individual pharmacokinetic parameters are expressed as means±s.d.
